# Supplementary material for: Implications of Cardioprotective Assumptions for National Drinking Guidelines and Alcohol Harm Monitoring Systems
Source: Int J Environ Res Public Health. 2019 Dec 6;16(24):4956. doi: 10.3390/ijerph16244956 (PMC6950575; doi:10.3390/ijerph16244956)
Supplement: Supplementary file 1 [file ijerph-16-04956-s001.pdf]

Supplementary Table S1: Alcohol-related deaths and condition weights, by condition and category, Australia and Canada, 2015

**Health condition**	**Australia**		**Canada**	
	**Deaths**	**Weight**	**Deaths**	**Weight**
Breast cancer	2,759	5.4%	5,188	4.9%
Colon and rectum cancer	4,061	8.0%	8,468	8.1%
Larynx cancer	206	0.4%	408	0.4%
Liver cancer	1,686	3.3%	2,900	2.8%
Esophageal cancer	1,246	2.4%	1,956	1.9%
Lip and oral cavity cancer	653	1.3%	950	0.9%
Atrial fibrillation and flutter	2,026	4.0%	3,737	3.6%
Hemorrhagic stroke	2,336	4.6%	3,775	3.6%
Hypertensive heart disease	2,164	4.3%	3,371	3.2%
Ischemic heart disease	18,435	36.2%	34,030	32.4%
Ischemic stroke	1,783	3.5%	9,912	9.4%
Diabetes mellitus	2,015	4.0%	6,808	6.5%
Cirrhosis	1,369	2.7%	3,169	3.0%
Pancreatitis	182	0.4%	449	0.4%
Tuberculosis	44	0.1%	107	0.1%
Lower respiratory infections	3,261	6.4%	7,732	7.4%
Epilepsy	243	0.5%	265	0.3%
Interpersonal violence	190	0.4%	437	0.4%
Self-harm	2,747	5.4%	4,349	4.1%
Transport injuries	1,015	2.0%	1,877	1.8%
Unintentional injuries	2,441	4.8%	5,175	4.9%
**Total**	**50,862**	**100.0%**	**105,063**	**100.0%**

**Health category**	**Australia**		**Canada**	
	**Deaths**	**Weight**	**Deaths**	**Weight**
Cancer	10,611	20.9%	19,870	18.9%
Cardiovascular conditions	26,744	52.6%	54,825	52.2%
Diabetes	2,015	4.0%	6,808	6.5%
Digestive conditions	1,551	3.0%	3,618	3.4%
Infectious diseases	3,305	6.5%	7,839	7.5%
Neuropsychiatric conditions	243	0.5%	265	0.3%
Injuries	6,393	12.6%	11,838	11.3%
**Total**	**50,862**	**100.0%**	**105,063**	**100.0%**

Notes: Only partially-attributable alcohol-related conditions are included in weighted RR functions.

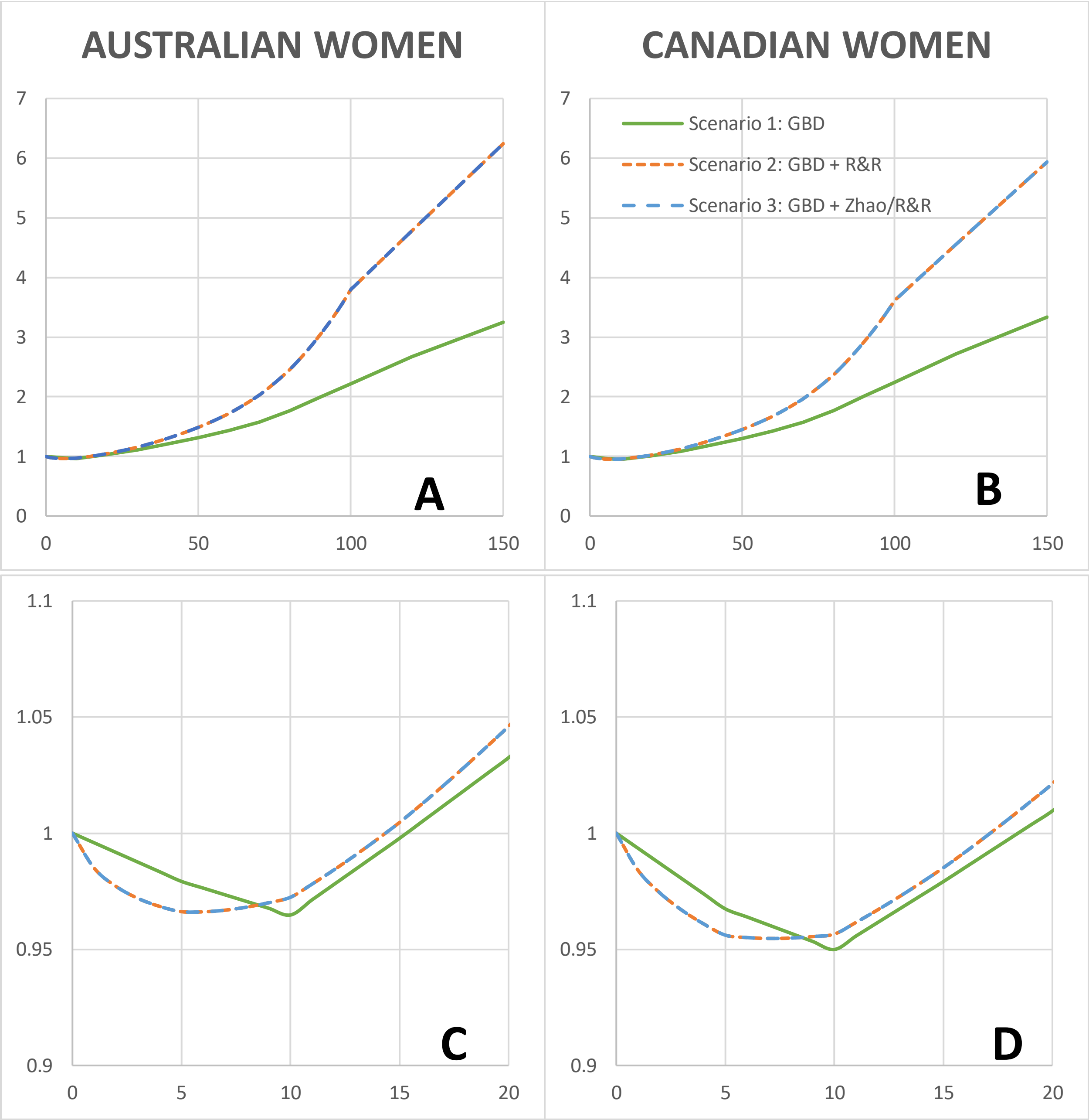

Supplemental Figure S1. Weighted relative risk functions, women, by cardioprotective scenario, Australia and Canada, 2015. Panel A: Australia, Net harm, Women; Panel B: Canada, Net harm, Women
Panel C: Same functions as in Panel A, magnified to a range of 0-20 g/day for resolution
Panel D: Same functions as in Panel B, magnified to a range of 0-20 g/day for resolution
y-axis: relative risk; x-axis: average daily alcohol consumption in grams ethanol/day
Note: For women, Scenarios 2 and 3 use the same IHD risk function (R&R) and so are corresponding functions are identical.

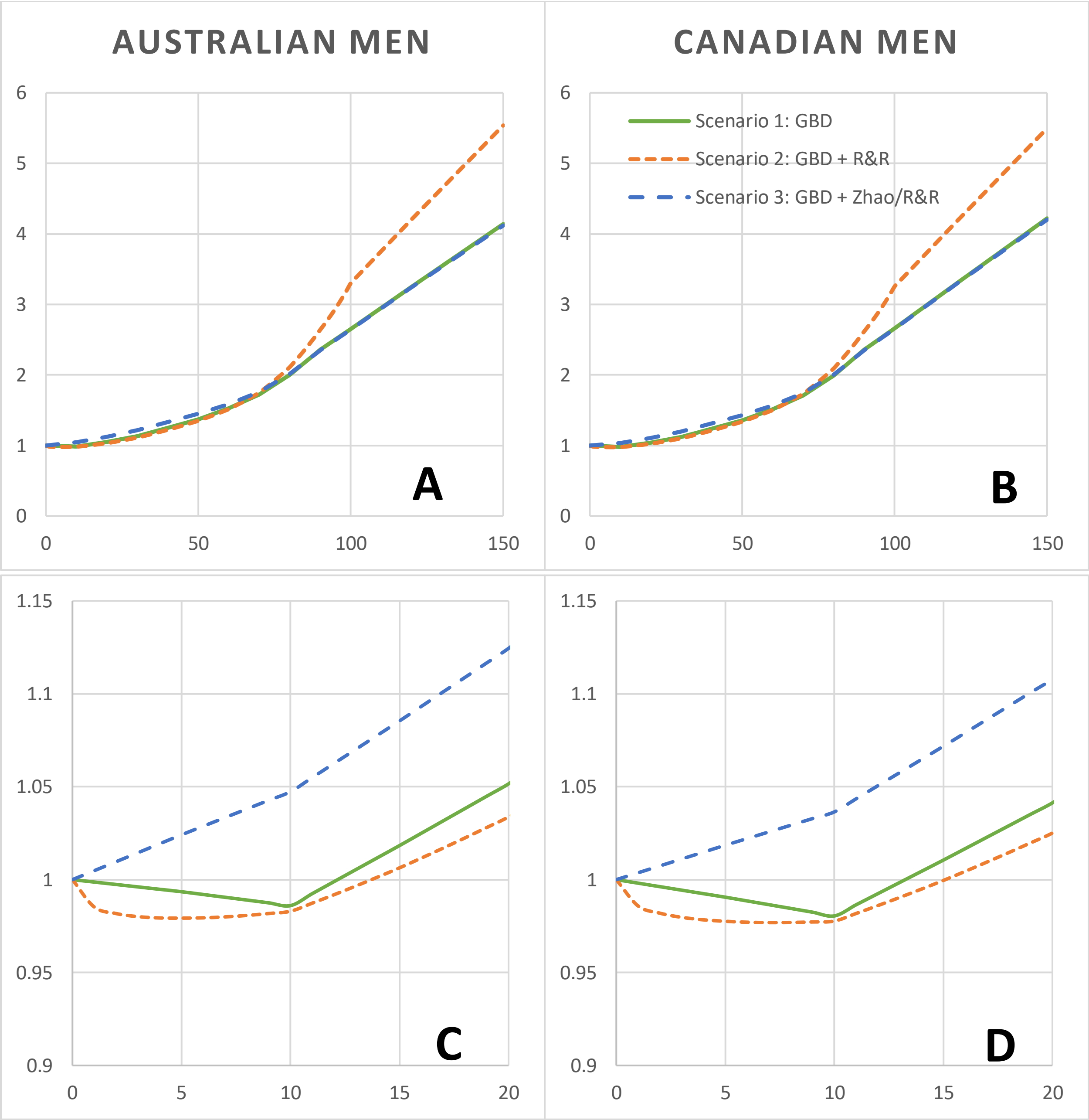

Supplemental Figure S2. Weighted relative risk functions, men, by cardioprotective scenario, Australia and Canada, 2015. Panel A: Australia, Net harm, Men; Panel B: Canada, Net harm, Men
Panel C: Same functions as in Panel A, magnified to a range of 0-20 g/day for resolution
Panel D: Same functions as in Panel B, magnified to a range of 0-20 g/day for resolution
y-axis: relative risk; x-axis: average daily alcohol consumption in grams ethanol/day

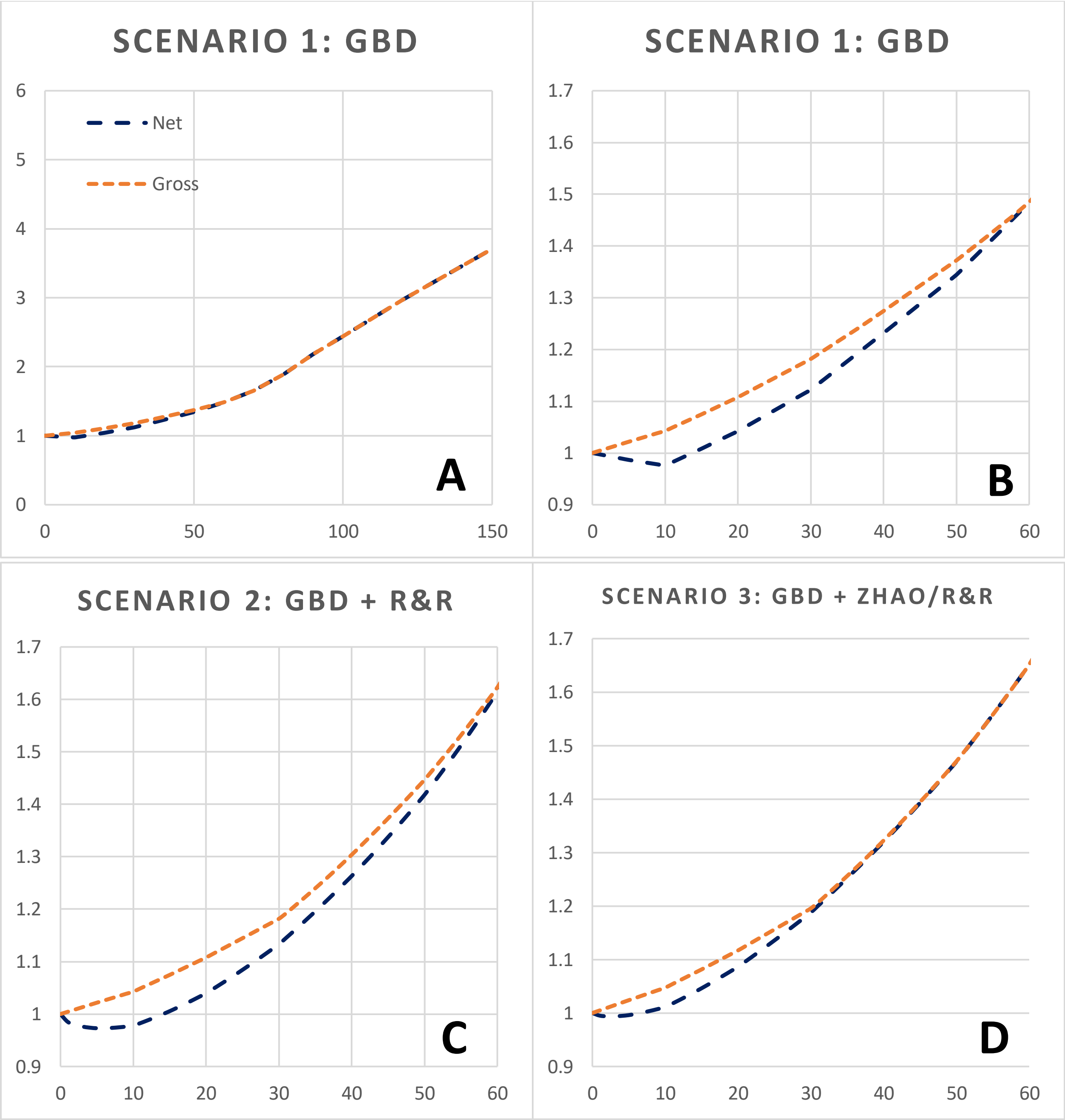

Supplemental Figure S3. Weighted relative risk functions, by harms (net vs. gross) and cardioprotective scenario, Australia, 2015

All Panels: Australia, genders combined, 2015.

Panel A: Cardioprotective scenario 1: GBD, at full scale to emphasize collapsing of net & gross harms at high levels of exposure;

Panel B: Same functions as in Panel A, magnified to a range of 0-60 g/day for resolution;

Panel C: Cardioprotective scenario 2: GBD + R&R, magnified to a range of 0-60 g/day for resolution

Panel D: Cardioprotective scenario 3: GBD + Zhao / R&R, magnified to a range of 0-60 g/day for resolution

y-axis: relative risk; x-axis: average daily alcohol consumption in grams ethanol/day

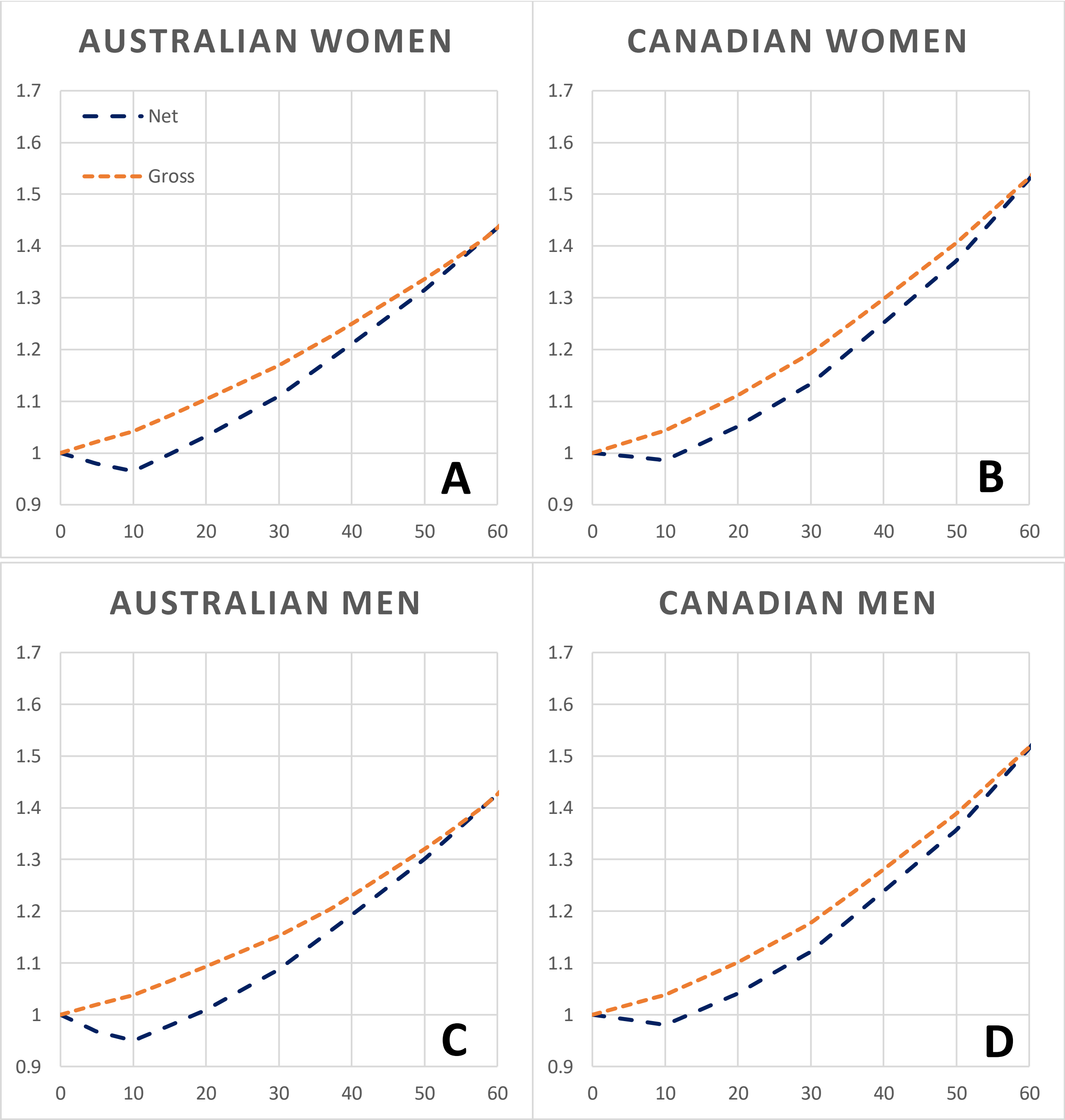

Supplemental Figure S4. Weighted relative risk functions, cardioprotective Scenario 1, by harms (net vs. gross), Australia and Canada, 2015;
Panel A: Australia, women, cardioprotective scenario 1: GBD;
Panel B: Canada, women, cardioprotective scenario 1: GBD;
Panel C: Australia, men, cardioprotective scenario 1: GBD;
Panel D: Canada, men, cardioprotective scenario 1: GBD
y-axis: relative risk; x-axis: average daily alcohol consumption in grams ethanol/day
